# Ultrashort Versus 1-Year Dual Antiplatelet Therapy Following Percutaneous Coronary Intervention: Meta-analysis of Randomized Controlled Trials

**DOI:** 10.1016/j.jscai.2024.102496

**Published:** 2025-02-18

**Authors:** Sheriff N. Dodoo, Benedicta Arhinful, Sammudeen Ibrahim, Olayiwola Bolaji, Afia S. Dodoo, Tracy Aggrey-Ansong, Vedang Bhavsar, Ugochukwu Egolum, Nima Ghasemzadeh, Ronnie Ramadan, Zachary H. George, Uzoma Ibebuogu, Habib Samady

**Affiliations:** a Georgia Heart Institute, Northeast Georgia Medical Center, Gainesville, Georgia; b Department of Internal Medicine, Howard University College of Medicine, Washington, DC; c Department of Internal Medicine, Piedmont Athens Regional Medical Center, Athens, Georgia; d Department of Cardio-Oncology, Memorial Sloan Kettering Cancer Center, New York, New York; e College of Pharmacy, Mercer University, Atlanta, Georgia; f Morrissey College of Arts and Sciences, Boston College, Chestnut Hill, Boston, Massachusetts; g Division of Cardiovascular Medicine, Department of Medicine, University of Tennessee Health Science Center, Memphis, Tennessee

**Keywords:** antiplatelet therapy, coronary, intervention

## Abstract

**Background:**

Dual antiplatelet therapy (DAPT) with aspirin and a P2Y12 receptor antagonist is the standard antithrombotic therapy after percutaneous coronary intervention (PCI); however, the optimal duration of this treatment remains a topic of ongoing debate. This study aimed to assess the clinical utility of an ultrashort dual antiplatelet therapy (US-DAPT) regimen (≤1 month) compared with standard DAPT (≥6 months) after PCI. In addition, the outcomes of choosing single antiplatelet therapy after US-DAPT, either clopidogrel or ticagrelor, were also analyzed.

**Methods:**

We queried MEDLINE, Cochrane Central Registry of Controlled Trials, Embase, and ClinicalTrials.gov databases from their commencement to May 2024 for all randomized controlled trials (RCTs) that directly compared US-DAPT (≤1 month) with standard therapy (≥6 months). The primary end point was net adverse clinical events (NACE), defined as a composite of major adverse cardiovascular or cerebrovascular events (MACCE) and clinically relevant bleeding (CRB).

**Results:**

Seven RCTs were included in the analysis, comprising 34,774 patients (US-DAPT, n = 17,383; standard therapy, n = 17,391) who were enrolled with a mean age of 67 ± 10 years and 74.7% male. US-DAPT was associated with a 20% lower risk of NACE (OR, 0.80; 95% CI, 0.68-0.94; *P* = .006; *I*^2^ = 74%) and 47% reduction in CRB (OR, 0.53; 95% CI, 0.37-0.75; *P* < .001; *I*^2^ = 77%) compared with standard therapy at 12 months. Similarly, US-DAPT was associated with statistically significant reduction in all-cause mortality (OR, 0.88; 95% CI, 0.77-0.99; *P* = .04; *I*^2^ = 0%) and TVR (OR, 0.87; 95% CI, 0.78-0.98; *P* = .02; *I*^2^ = 41%) However, no significant difference in MACCE, all-cause mortality, cardiovascular disease–related deaths, MI, stroke, MI, TVR, and ST was observed.

**Conclusions:**

In patients undergoing PCI, US-DAPT was associated with lower NACEs and bleeding risk without increasing the occurrence of ischemic events, including ST and MI, when compared with at least 6 months of DAPT, irrespective of the choice of single antiplatelet therapy, whether clopidogrel or ticagrelor, following DAPT.

Dual antiplatelet therapy (DAPT) using aspirin and a P2Y12 receptor antagonist is the standard of care for patients who undergo implantation of drug-eluting stents (DES) for coronary artery disease.^1^ DAPT after placement of coronary stents has anti-ischemic benefits, including prevention of stent thrombosis (ST), myocardial infarction (MI), and target-vessel revascularization (TVR).^2^ However, DAPT is associated with increased risks of clinically relevant bleeding (CRB) episodes, including intracranial hemorrhage and death.^3^ Interestingly, while the risk of ST is highest in the early postprocedural period after percutaneous coronary intervention (PCI), the risk of bleeding events increases with more prolonged DAPT.^4^^,^^5^ Therefore, the optimal duration of DAPT is a balance of the anti-ischemic benefits and the bleeding prevention.

Current practice guidelines recommend that a short duration of DAPT after percutaneous revascularization in patients with stable ischemic heart disease is reasonable to reduce the risk of bleeding events.^1^ The guidelines also suggest that some select patients may be transitioned to a single P2Y12 receptor antagonist after 1 to 3 months of DAPT, after consideration of ischemic and bleeding risks.^1^ The optimal length of DAPT following PCI is an ongoing debate. Several studies have evaluated the clinical utility of short duration (≤6 months) of DAPT.^6^, ^7^, ^8^, ^9^ However, whether short DAPT offers sufficient protection against thrombotic events while reducing bleeding episodes is still unclear. Additionally, the choice of the impact of single antiplatelet agents, whether clopidogrel or ticagrelor, following the initial DAPT, on the assessed end points of interest is unknown.

We performed this meta-analysis to investigate the clinical benefits of ultrashort dual antiplatelet therapy (US-DAPT; ≤1 month) duration after implantation of coronary stents in patients with coronary artery disease, compared with standard (≥6 months) DAPT. Additionally, we evaluated the risks of bleeding and ischemic events of the choice of a P2Y12 receptor antagonist following the initial DAPT, comparing clopidogrel with ticagrelor.

## Materials and methods

This analysis complied with the Preferred Reporting Items for Systematic Reviews and Meta-Analyses guidelines.^10^ The protocol for this meta-analysis was registered to the Prospective Register of Systematic Reviews (CRD 42024558928). The institutional review board deemed the study exempt since the data used for the final analysis are publicly available and deidentified. Upon reasonable request, data supporting this study’s findings are available from the corresponding author.

### Data sources and study selection

Two authors (S.N.D. and S.I.) queried PubMed, Cochrane Central Registry of Controlled Trials, EMBASE, and ClinicalTrials.gov databases from their commencement to May 2024 for all randomized controlled trials (RCTs) that directly compared US-DAPT (≤1 month) with standard therapy (≥6 months). The following keywords were used for the search: antiplatelet therapy, clopidogrel, prasugrel, ticagrelor, percutaneous coronary intervention, and drug-eluting stent. These authors independently reviewed the studies for eligibility according to the prespecified criteria. All discrepancies were resolved by a third author (U.E.).

### Study selection and quality assessment

The following were inclusion criteria. Studies that met the prespecified eligibility criteria were studies published in peer-reviewed journals. The included studies were RCTs that directly compared US-DAPT (≤1 month) with standard therapy (≥6 months). The studies used in that analysis included those that used contemporary-generation DES. Trials that investigated clinical outcomes of patients treated with bare-metal stents were excluded. Freedom from bias was independently assessed for the included studies in compliance with the Cochrane Collaboration 2.0 software^11^ (Supplemental Appendix).

### Data extraction and outcome variables

The primary end point was net adverse clinical events (NACE), defined as a composite of major adverse cardiovascular or cerebrovascular events (MACCE) and CRB. MACCE was defined as a composite of all-cause mortality, MI, stroke, ST, and TVR. While the definition of major and minor bleeding events of the included studies was not homogenous, to improve the sample size and power of the analysis, CRB was used to define the safety end point of this study. CRB was a composite of major or minor bleeding, defined as Bleeding Academic Research Consortium (BARC) type 2, 3, 4, or 5, as well as Thrombolysis in Myocardial Infarction major or minor bleeding. The secondary end points included MACCE, CRB, all-cause mortality, cardiac mortality, MI, stroke, ST, and TVR.

### Data synthesis and analysis

The Mantel-Haenszel odds ratio (OR) with a 95% CI was analyzed using a random-effects model to account for significant heterogeneity between the included studies. A *P* <.05 was considered statistically significant without adjustment for multiplicity. Heterogeneity was adjudicated significant if the *I*^2^ statistics was ≥50%; otherwise, the fixed-effects model was used. The study variance was calculated in compliance with the DerSimonian-Laird method.

The study treatment effect of the single P2Y12 receptor antagonist on the end points of interests—ticagrelor or clopidogrel used after DAPT for the ultrashort (≤1 month) and standard duration (≥6 months) was assessed by pooling all included trials in a subgroup analysis. All statistical analyses were done using Review Manager version 5.4.1 (The Nordic Cochrane Center, the Cochrane Collaboration, 2014).

## Results

**Figure 1:**
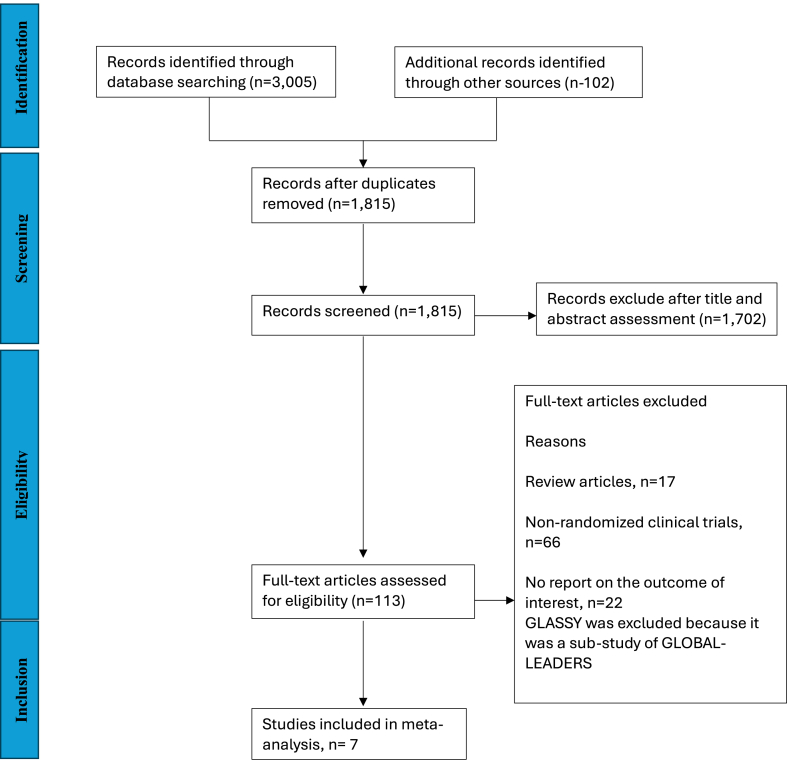
**PRISMA schematicflow diagram.** PRISMA, Preferred Reporting Items for Systematic Reviews and Meta-Analyses.

The flow diagram for the RCT selection is shown in Figure 1. Following adjudication with the application of the eligibility criteria, 7 RCTs were included in the analysis. A total of 34,774 patients were enrolled in the study and randomized to either US-DAPT (≤1 month, n = 17,383) or standard therapy (≥6 months, n = 17,391). Among the population enrolled, 15.9% presented with ST-segment elevation myocardial infarction (STEMI), 19.5% presented with non-STEMI, 16.8% presented with unstable angina (UA), and 47.8% had chronic coronary syndrome (CCS) requiring PCI. A summary of the baseline characteristics of the study population is shown in Supplemental Table S1.

### Clinical end points

**Figure 2:**
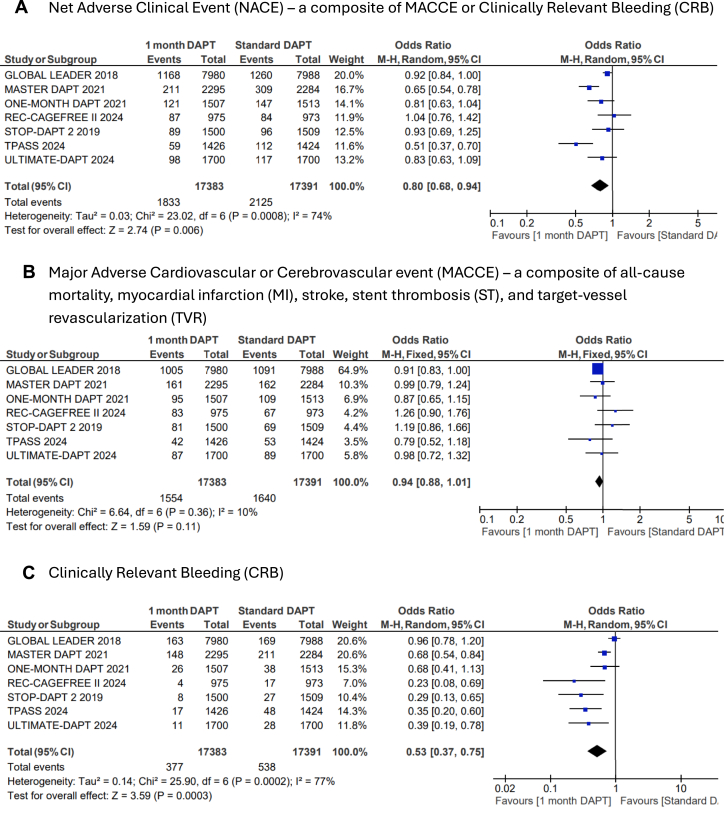

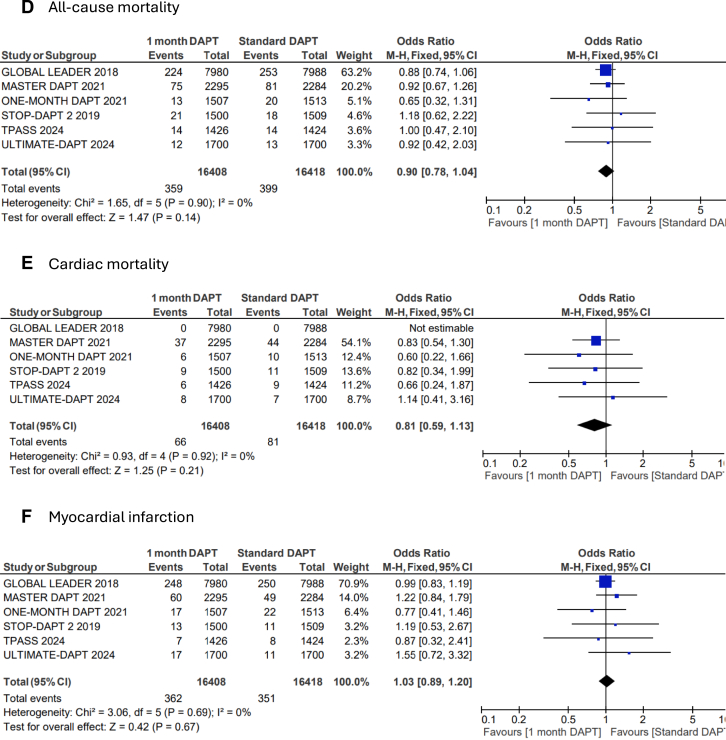

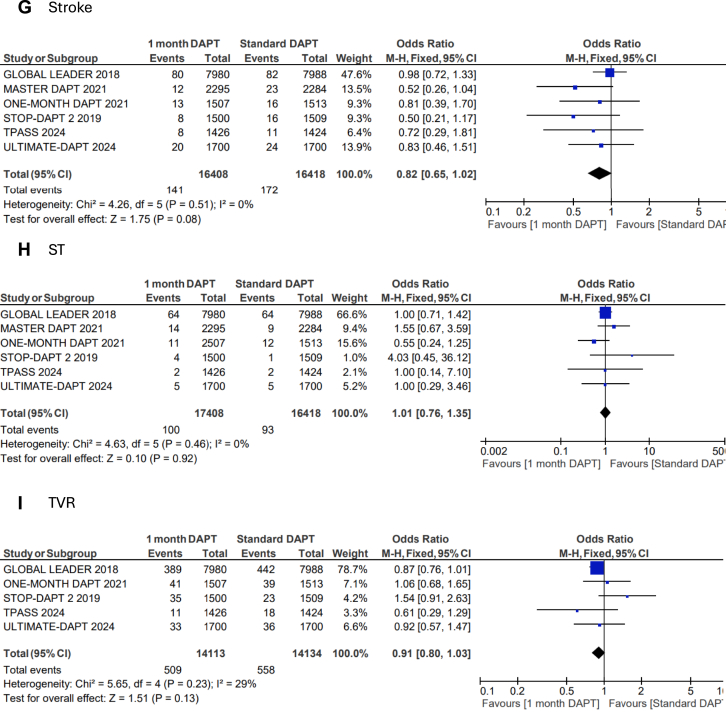
**Forest plot showing the assessed end points of interest comparing ultrashort (≤1 month) dual antiplatelet therapy (DAPT), followed by single antiplatelet therapy (clopidogrel or ticagrelor) vs standard (≥6 months) DAPT.** The odds ratios for the random effects are presented. (**A**) Net adverse clinical event (NACE)—a composite of major adverse cardiovascular or cerebrovascular event (MACCE) or clinically relevant bleeding (CRB); (**B**) MACCE—a composite of all-cause mortality, myocardial infarction (MI), stroke, stent thrombosis (ST), and target-vessel revascularization (TVR); (**C**) CRB; (**D**) all-cause mortality; (**E**) cardiac disease–related mortality; (**F**) MI; (**G**) stroke; (**H**) ST; (**I**) TVR.

US-DAPT was associated with 20% lower odds of the primary end point of NACE when compared with standard therapy (OR, 0.80; 95% CI, 0.68-0.94; *P* = .006; *I*^2^= 74%) (Figure 2A). There were similar odds of the secondary end point of MACCE when US-DAPT was compared with standard therapy (OR, 0.94; 95% CI, 0.88-1.01; *P* = .11; *I*^2^ = 10%) (Figure 2B). US-DAPT was associated with 47% lower odds of secondary end point of CRB (OR, 0.53; 95% CI, 0.37-0.75; *P* < .001) (Figure 2C) compared with standard therapy.

However, there was no significant difference in the odds of all-cause mortality (OR, 0.90; 95% CI, 0.78-1.04; *P* = .14) (Figure 2D), cardiovascular disease–related deaths (OR, 0.81; 95% CI, 0.59-1.13; *P* = .21) (Figure 2E), MI (OR, 1.03; 95% CI, 0.89-1.20; *P* = .67) (Figure 2F), stroke (OR, 0.82; 95% CI, 0.65-1.02; *P* = .08) (Figure 2G), TVR (OR, 0.91; 95% CI, 0.80-1.03; *P* = .13) (Figure 2I), and ST (OR, 1.01; 95% CI, 0.76-1.35; *P* = .39) (Figure 2H) when US-DAPT was compared with standard therapy (Central Illustration).

**Central Illustration:**
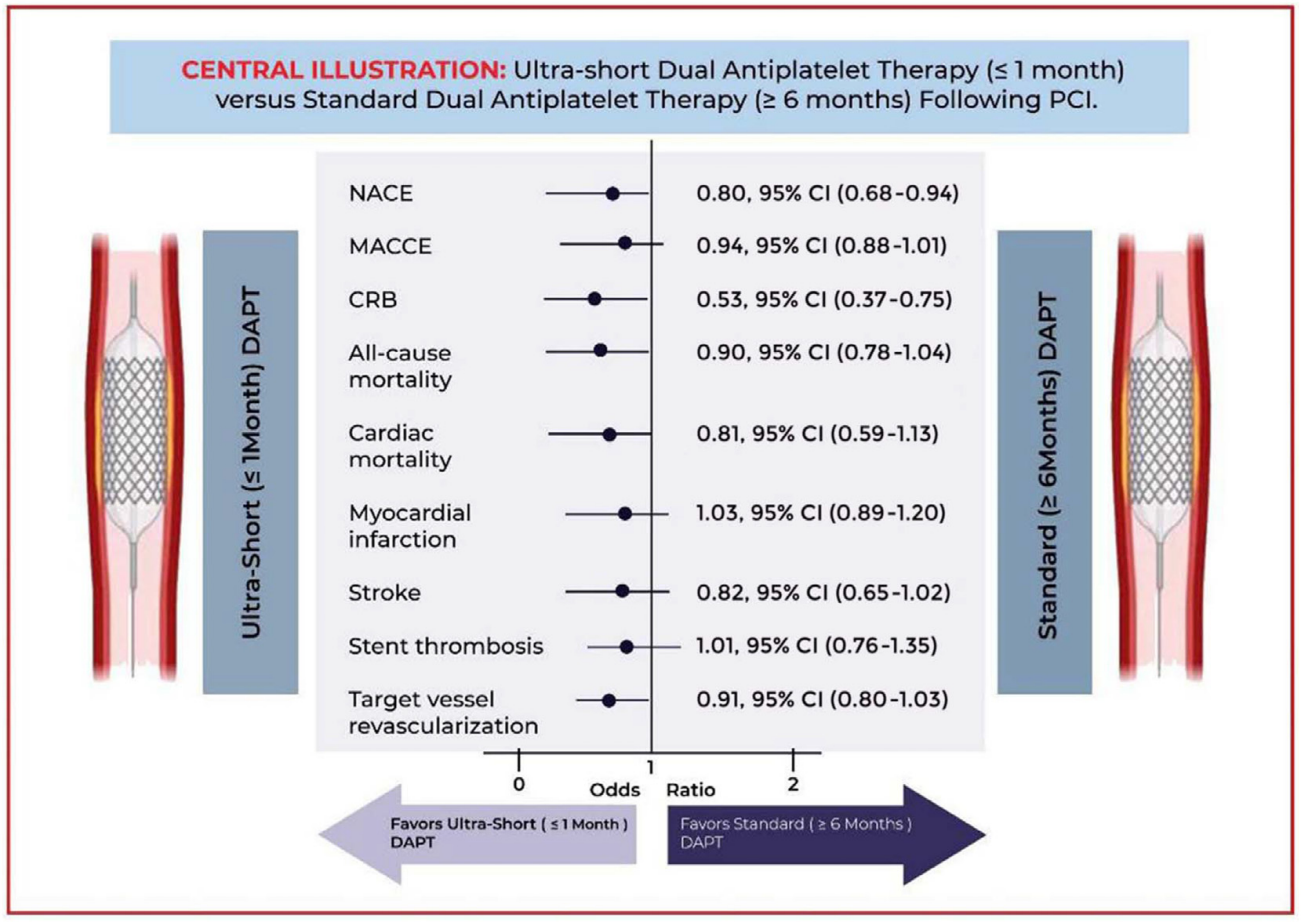
**Clinical utility and safety of ultrashort dual antiplatelet therapy (DAPT) following percutaneous coronary intervention.** CRB, clinically relevant bleeding; MACCE, major adverse cardiovascular or cerebrovascular event; NACE, net adverse clinical event.

### Treatment effect of ticagrelor monotherapy and clopidogrel monotherapy after initial US-DAPT

**Figure 3:**
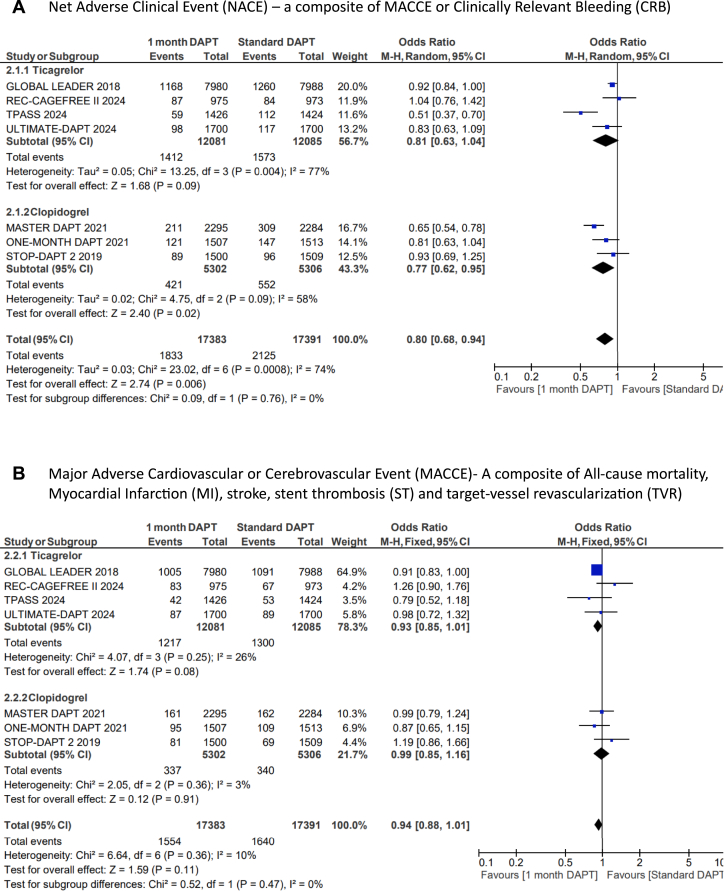

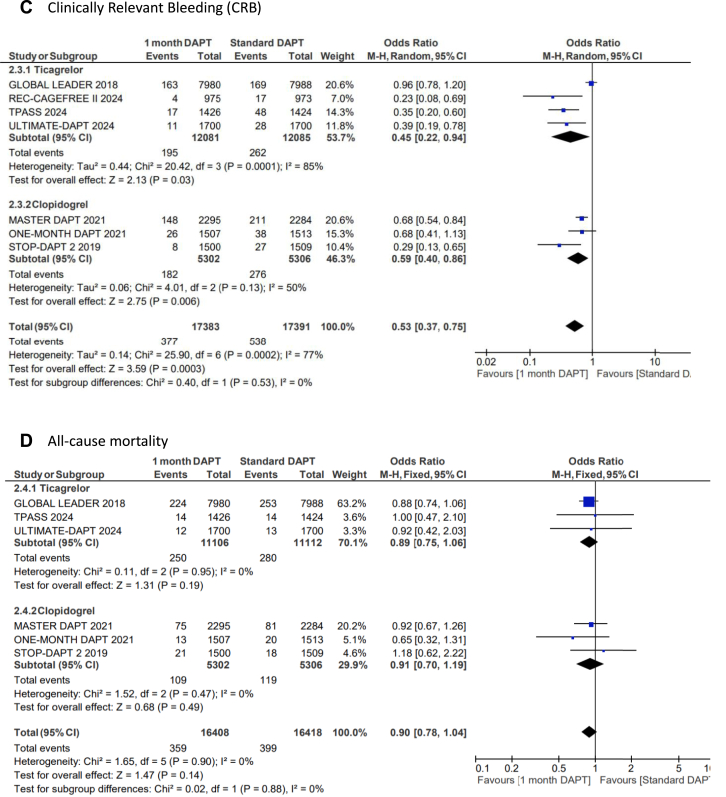

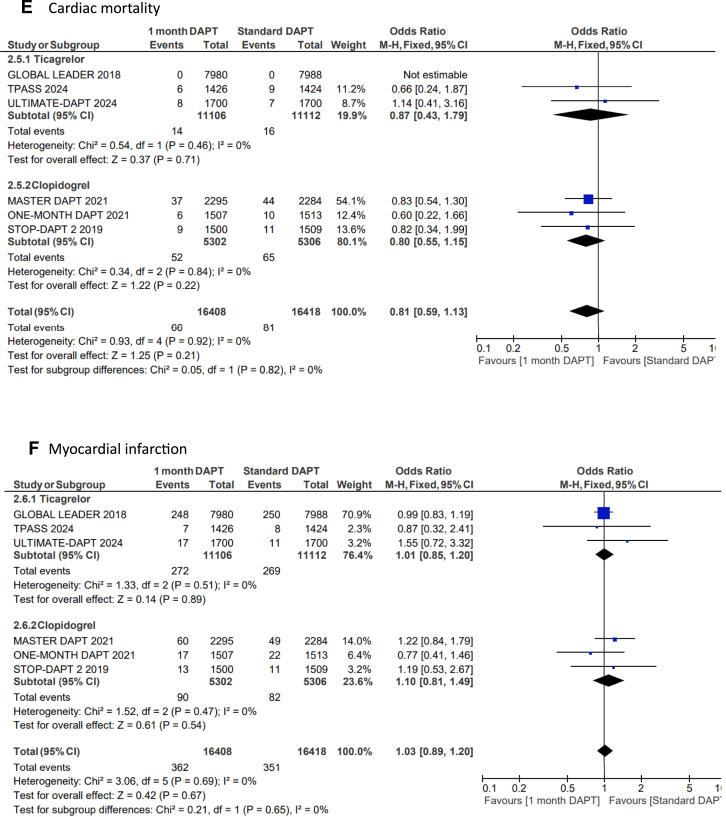

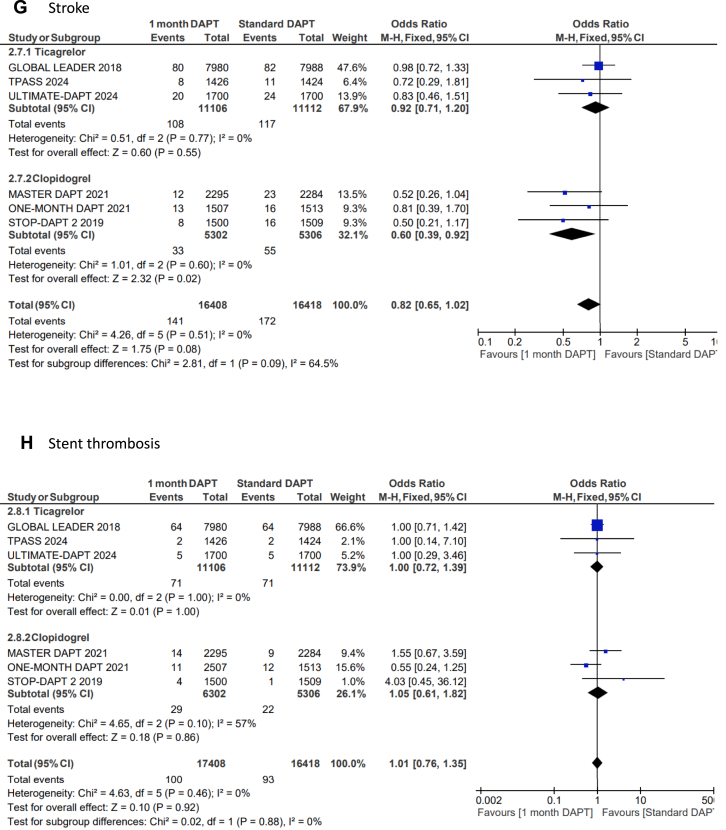

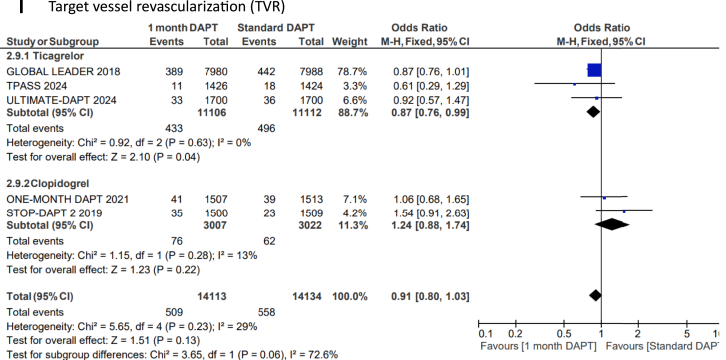
**Forest plots showing the assessed end points of interest comparing ultrashort (≤1 month) dual antiplatelet therapy (DAPT) versus standard (≥6 months) DAPT with the population segregated by the choice of single antiplatelet therapy following DAPT, whether clopidogrel or ticagrelor.** The odds ratios for the random effects are presented. (**A**) Net adverse clinical event (NACE)—a composite of major adverse cardiovascular or cerebrovascular event (MACCE) or clinically relevant bleeding (CRB); (**B**) MACCE—a composite of all-cause mortality, myocardial infarction (MI), stroke, stent thrombosis (ST), and target-vessel revascularization (TVR); (**C**) CRB; (**D**) all-cause mortality; (**E**) cardiac disease–related mortality; (**F**) MI; (**G**) stroke; (**H**) ST; (**I**) TVR.

In a separate subgroup analysis, the treatment effect of ticagrelor monotherapy following the initial US-DAPT on the primary and secondary end points was analyzed in 4 RCTs, compared with standard therapy (≥6 months). This subgroup analysis was repeated using 3 RCTs that used clopidogrel monotherapy following the initial US-DAPT, compared with standard therapy. There were no significant differences between ticagrelor and clopidogrel in all outcomes of interest measured (Figure 3A-I).

## Discussion

Previous studies have assessed the efficacy of 3 months of DAPT^12^, ^13^, ^14^ and similar durations of DAPT specifically within the ACS population^15^ and those with high bleeding risks.^16^^,^^17^ However, there is a paucity of studies exploring shorter durations of DAPT in ≤1 month. This meta-analysis, to our knowledge, the largest study thus far, fills this gap by specifically examining the safety and clinical utility of a 1-month DAPT regimen.

In this contemporary systemic review and meta-analysis of 7 RCTs involving 34,774 all-comers with coronary artery disease, presenting with STEMI, non-STEMI, UA, and CCS, who underwent PCI and subsequently received US-DAPT (≤1 month) followed by a single P2Y12 receptor antagonist or standard DAPT for at least 6 months, we reported several significant findings.

First, the use of US-DAPT (≤1 month) followed by a single P2Y12 receptor antagonist, whether ticagrelor or clopidogrel, was associated with lower NACE, a composite of MACCE or CRB, when compared with standard DAPT of at least 6 months, following PCI. Second, while there is no difference in MACCE comparing US-DAPT (≤1 month) with standard DAPT (≥6 months), US-DAPT for ≤1 month followed by a single P2Y12 receptor antagonist, whether ticagrelor or clopidogrel, was associated with lower CRB. Third, the use of US-DAPT (≤1 month) after PCI confined survival benefits with lower all-cause mortality, compared with standard therapy of DAPT (≥6 months). Fourth, TVR was lower in the patients who received US-DAPT (≤1 month) followed by a single P2Y12 receptor antagonist (whether ticagrelor or clopidogrel) compared with that in those who had standard DAPT for at least 12 months. Fifth, there was no significant difference in the odds of cardiovascular disease–related mortality, MI, stroke, and ST detected with therapeutic strategies of US-DAPT (≤1 month) versus standard therapy of DAPT for at least 6 months after PCI. Finally, the use of ticagrelor in the US-DAPT strategy was associated with a reduction in TVR compared with clopidogrel following PCI.

The MASTER-DAPT trial was one of the initial RCTs that demonstrated that among high bleeding risk patients, abbreviated DAPT of 1 month was noninferior to standard DAPT for at least 6 months following placement of bioresorbable polymer-based sirolimus-eluting stents for NACE or MACCE but superior for major CRB.^8^ In this study, as with MASTER-DAPT, US-DAPT was associated with lower odds of NACE, which was driven by lower odds of CRB, but it had similar odds of MACCE when compared with at least 6 months of DAPT after PCI. It is important to note that while the MASTER-DAPT trial included patients with high bleeding risks, our contemporary meta-analysis did stratify patients based on their bleeding risks. In consonance with the findings of this contemporary meta-analysis, results of the STOPDAPT-2 trial showed that in a cohort of 3045 patients, not selected based on their bleeding risks, 1 month of DAPT followed by clopidogrel when compared with 12 months of DAPT was associated with a lower rate of a composite of cardiovascular and bleeding events, meeting criteria for both noninferiority and superiority.^18^

Recently, Hong et al^19^ reported findings of the pivotal T-PASS trial. This randomized noninferiority study randomized 2850 patients to 1 month of DAPT followed by ticagrelor monotherapy or 12 months of ticagrelor-based DAPT. They found that the 1-month DAPT followed by the ticagrelor monotherapy arm was both superior and noninferior to the 12-month DAPT strategy with 46% risk reduction of a composite of all-cause death, MI, definite or probable ST, stroke, and major bleeding events at 1 year after the index procedure. These findings were reflected in our meta-analysis: 20% reduction in odds of the NACEs (a composite of MACCE or CRB).^19^

Additionally, results of the ULTIMATE-DAPT trial showed that in patients with an acute coronary syndrome who had PCI with contemporary DES and remained event-free for 1 month on DAPT, treatment with ticagrelor alone between month 1 and month 12 after the index procedure had a lower rate of CRB and a similar rate of MACCE compared with 12 months of ticagrelor-based DAPT.^20^ The treatment effect of US-DAPT (≤1 month) followed by a single P2Y12 receptor antagonist was similarly reflected in our meta-analysis as in the ULTIMATE-DAPT trial.

The newer generation of DES platforms with biocompatible polymers and thinner strut cells has shown lower rates of ischemic events, including major adverse cardiovascular events, in patients without or with high bleeding risks, despite relatively short DAPT strategies, compared with bare-metal stents.^20^, ^21^, ^22^, ^23^ These intriguing results echoed the findings of this contemporary meta-analysis, as all included RCTs used second-generation DES with biocompatible polymers with similar odds of lower MACCE and ST. Hence, US-DAPT (<1 month) followed by a single P2Y12 receptor antagonist strategy should be applied to patients who received second-generation DES platforms following a coronary-related event.

### Limitations

The findings of this contemporary meta-analysis were made in the context of several significant limitations. First, like all meta-analyses, this study cannot overcome the inherent limitations of the individual RCT included in the final analysis. The lack of a placebo control in the design of the included RCT may be a challenge. However, the included RCTs were all blindly adjudicated by an independent event investigator to mitigate the introduction of performance bias.

Second, the choice of a single P2Y12 receptor antagonist following the US-DAPT strategy was not protocolized and randomized. That choice of P2Y12 receptor antagonist was based on the treating physician’s preference. A subgroup analysis was done to evaluate the treatment effect of the choice of P2Y12 receptor antagonist on the outcomes of interest. This analysis showed that the choice of P2Y12 receptor antagonist following the initial US-DAPT was homogenous between the 2 comparative arms. However, the effects of unmeasured confounders could not be assessed.

Third, the heterogeneity of the definition of major or minor bleeding events adopted by the individual RCTs included in the final analysis was a potential source of comparator bias. To mitigate this limitation, the research protocol was designed to define CRB in agreement with BARC type 2, 3, 4, or 5 as well as thrombolysis in myocardial infarction major or minor bleeding, in the RCT that did not provide information on the BARC bleeding type. This approach provided the opportunity to best approximate bleeding event definition in real-world situations and in the context of clinical research such as this contemporary meta-analysis.

Fourth, 3 trials (MASTER-DAPT, STOP DAPT 2, and ULTIMATE-DAPT) included only patients who remained event free at 1 month or at the time of hospital discharge, which may introduce selection bias. Unfortunately, this limitation cannot be adequately addressed in a trial-level meta-analysis.^8^^,^^18^^,^^20^ Finally, the treatment effects of the complex PCI, including bifurcation and left main coronary stenting, were not assessed. However, previous studies have shown promising results,^24^^,^^25^ which is beyond the scope of this contemporary meta-analysis.

## Conclusions

In patients with coronary artery disease presenting with STEMI, non-STEMI, UA, and CCS who underwent PCI, the use of US-DAPT for ≤1 month, followed by monotherapy with a P2Y12 antagonist, ticagrelor or clopidogrel, was associated lower NACE, compared with at least 6 months of DAPT. The net benefit of the US-DAPT was driven by a reduction in CRB. High-fidelity studies are needed to corroborate the clinical benefits of US-DAPT followed by monotherapy with a P2Y12 antagonist after PCI with the placement of DES. Future directions in research should compare the safety and efficacy of antiplatelet monotherapy, whether ticagrelor or clopidogrel, following abbreviated DAPT.
