# Supplementary material for: Ultrashort Versus 1-Year Dual Antiplatelet Therapy Following Percutaneous Coronary Intervention: Meta-analysis of Randomized Controlled Trials
Source: J Soc Cardiovasc Angiogr Interv. 2025 Feb 18;4(2):102496. doi: 10.1016/j.jscai.2024.102496 (PMC11916820; doi:10.1016/j.jscai.2024.102496)
Supplement: Supplementary Material [file mmc1.docx]

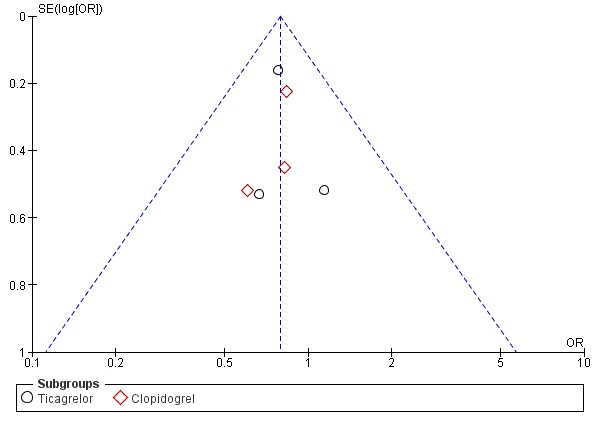


Funnel plot of comparison: 2 Ultra-short (1 month) versus Standard DAPT, outcome: 2.5 CV Mortality.


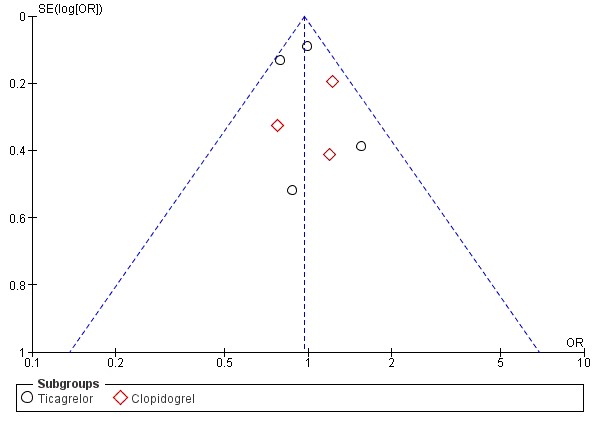


Funnel plot of comparison: 2 Ultra-short (1 month) versus Standard DAPT, outcome: 2.6 MI.


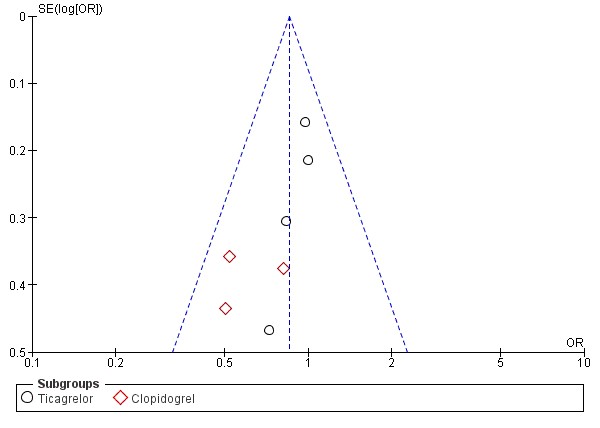


Funnel plot of comparison: 2 Ultra-short (1 month) versus Standard DAPT, outcome: 2.7 Stroke.


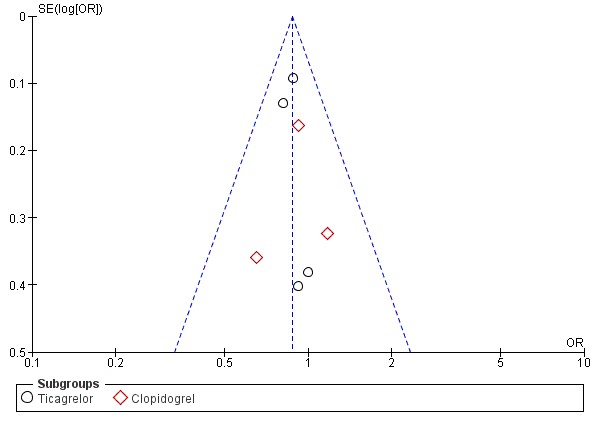


Funnel plot of comparison: 2 Ultra-short (1 month) versus Standard DAPT, outcome: 2.4 All-cause mortality.


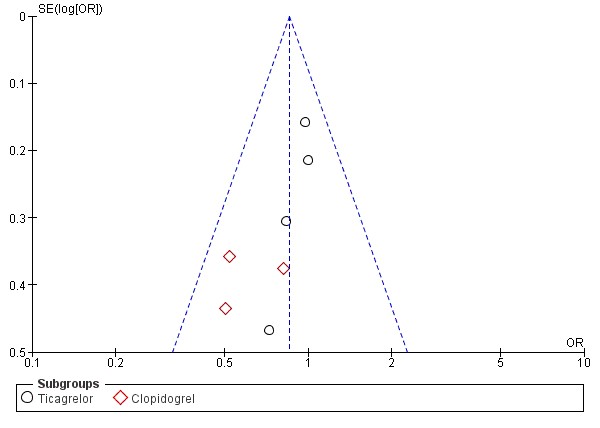


Funnel plot of comparison: 2 Ultra-short (1 month) versus Standard DAPT, outcome: 2.7 Stroke.


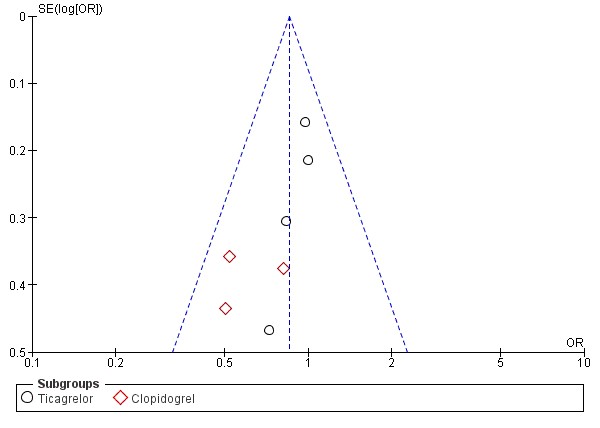


Funnel plot of comparison: 2 Ultra-short (1 month) versus Standard DAPT, outcome: 2.4 All-cause mortality.


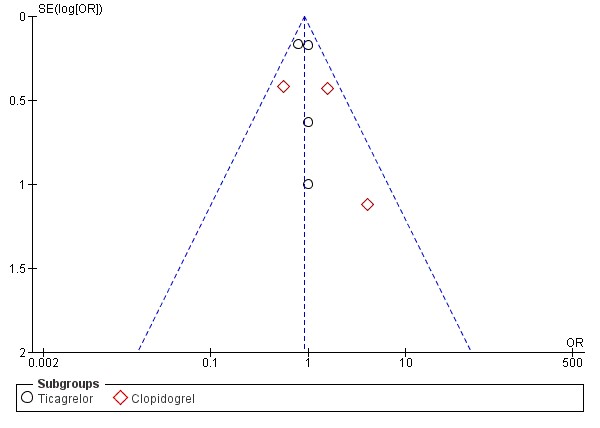


Funnel plot of comparison: 2 Ultra-short (1 month) versus Standard DAPT, outcome: 2.8 ST.


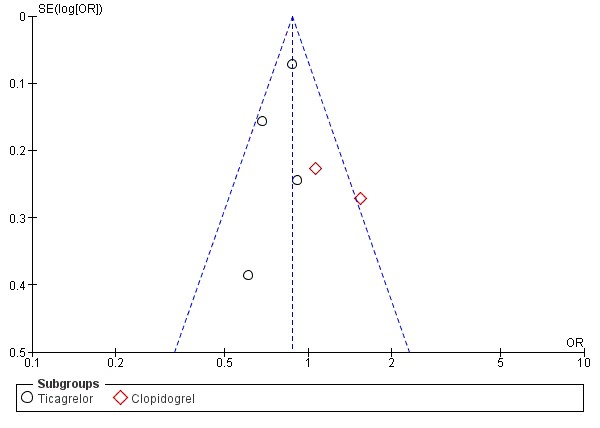


Funnel plot of comparison: 2 Ultra-short (1 month) versus Standard DAPT, outcome: 2.9 TVR.


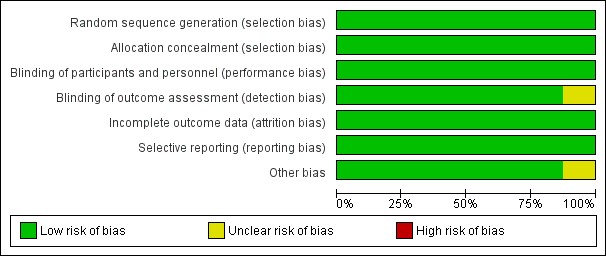


Risk of bias graph: review authors' judgements about each risk of bias item presented as percentages across all included studies.


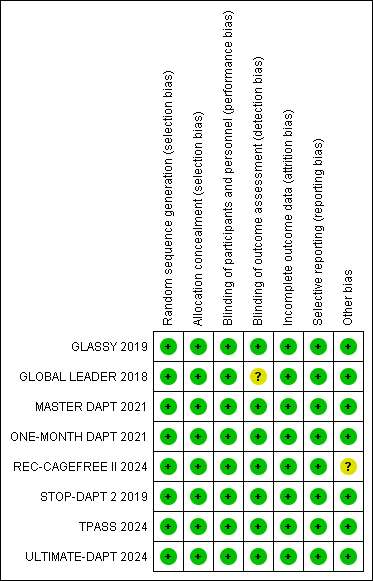


Risk of bias summary: review authors' judgements about each risk of bias item for each included study.
