# Supplementary material for: Ultrashort Versus 1-Year Dual Antiplatelet Therapy Following Percutaneous Coronary Intervention: Meta-analysis of Randomized Controlled Trials
Source: J Soc Cardiovasc Angiogr Interv. 2025 Feb 18;4(2):102496. doi: 10.1016/j.jscai.2024.102496 (PMC11916820; doi:10.1016/j.jscai.2024.102496)
Supplement: Supplementary Data [file mmc2.docx]

| **Study/Trial** | **GLOBAL-LEADER** | **MASTER-DAPT** | **ONE-MONTH DAPT** | **REC-CAGEFREE II** | **STOP-DAPT 2** | **TPASS** | **ULTIMATE-DAPT** |
| --- | --- | --- | --- | --- | --- | --- | --- |
| Year | 2018 | 2021 | 2021 | 2024 | 2019 | 2024 | 2024 |
| Design | open label, randomized  superiority trial | multicenter, randomized,  open-label, non-inferiority trial with sequential  superiority testing. | multicenter, randomized, open-label trial | Investigator initiated, prospective, open-label, multicenter, non-inferiority trial | Multicenter, open label, randomized clinical trial | randomized, open-label, non-inferiority trial | randomized, placebo-controlled, double-blind clinical trial |
| Region | Austria, Belgium, Germany, Netherlands, Italy, UK, Switzerland, Poland, Bulgaria | 30 Countries | South Korea | China | Japan | South Korea | China, Italy, Pakistan, and the UK. |
| Recruitment Period | Jul 2013 - Nov 2015 | Feb 2017 - Dec 2019 | Dec 2015 - Sep 2019 | Nov 2021 – March 2023 | Dec 2015 - Dec 2017 | NR | Sept 2019 - Oct 2022 |
| Follow-up | 30 days and  3, 6, 12, 18, and 24 months after the index procedure. | Follow-up visits occurred  at 60 days (within a window of ±14 days) and 150 days (±14-day window) after randomiza-  tion, preferably as on-site visits, and at 335 days (±14-day window) after randomization, exclusively as an on-site visit. | 12 months | N/A | 1 year | Follow-up visits: 1 to 4 weeks and 3, 6,  and 12 months after the index procedure. | Post-discharge visits after the initial percutaneous  coronary interventions were scheduled at 1, 4, 6,  and 12 months |
| Key Inclusion Criteria | 1. Age ≥18 years;  2. Patients with any clinical indication for percutaneous coronary intervention  3.Presence of one or more coronary artery stenosis of 50% or more in a native coronary artery or in a saphenous venous or arterial bypass conduit suitable for coronary stent implantation in a vessel with a reference vessel diameter of at least 2.25 millimeter. | Patients who had an acute or chronic coronary syndrome; had undergone suc-  cessful PCI for one or more coronary-artery stenoses with implantation of a biodegradable-  polymer sirolimus-eluting stent free from adverse cardiovascular events (including a new acute coronary syn-  drome, symptomatic restenosis, stent thrombosis, stroke, or any revascularization resulting in the prolonged use of dual antiplatelet therapy)  during the first month after the index PCI. | 1.Patients ≥19 years old  2. Patients with ischemic heart disease who are considered for treatment with PCI  3. Significant coronary de novo lesion | Patients with an indication for PCI due to acute coronary syndrome (including STEMI, NSTEMI, and unstable angina)  All intended target lesion(s) are successfully treated by PCI with only drug-coated balloon(s)  Patients who are able to complete the follow-up and compliant to the prescribed medication | Patients who have undergone PCI with the everolimus-eluting cobalt-chromium stent (CoCr-EES, XienceTM) and have not experienced major complications (death, MI, stroke, or major bleeding) during hospital stay for treatment  Patients who are capable of oral dual antiplatelet therapy consisting of aspirin and a P2Y12 receptor antagonist | 1. Patients ≥19 years  2. Patients who received new generation sirolimus-eluting (Osiro® series) stent implantation for treating ACS, including acute MI and unstable angina  3. Provision of informed consent | Remain event-free for 1 month on dual  antiplatelet therapy following percutaneous coronary intervention with contemporary drug-eluting stents.  Patients aged 18 years or older; had an acute  coronary syndrome (ie, unstable angina [angiography  showing a severely narrowed or ruptured plaque or  thrombotic lesion without cardiac biomarker elevation],  non-ST-segment elevation myocardial infarction  [NSTEMI], or ST-segment elevation myocardial infarction  [STEMI]) caused by a culprit lesion in an untreated  coronary artery segment, up to 30 days before  randomisation;18 and had an indication for percutaneous coronary intervention with a second-generation drug-  eluting stent |
| Key Exclusion Criteria | 1.Known intolerance to aspirin, P2Y12 receptor antagonists, bivalirudin, stainless steel or biolimus  2.Known intake of a strong cytochrome P3A4 inhibitor (eg, ketoconazole, clarithromycin, nefazodone, ritonavir, and atazanavir), as co-administration may lead to a substantial increase in exposure to ticagrelor  3.Use of fibrinolytic therapy within 24 hours of percutaneous coronary intervention  4.Known severe hepatic impairment  5.Planned coronary artery bypass grafting as a staged procedure (hybrid) within 12 months of the index procedure  6.Planned surgery within 12 months of percutaneous coronary intervention unless dual antiplatelet therapy is maintained throughout the peri-surgical period  7.Need for oral anti-coagulation therapy  8.PCI for a priori known stent thrombosis  9.Known overt major bleeding  10.Known history of intracranial hemorrhage  11. Known stroke from ischemic or unknown cause within last 30 days  12. Known pregnancy at time of randomization  13. Inability to provide informed consent  14.Currently participating in another trial before reaching primary endpoint | Implantation of a stent other than the Ultimaster stent within 6 months  before the index procedure, the implantation of  a bioresorbable scaffold at any time before the index procedure, and treatment for in-stent re-  stenosis or stent thrombosis. | acute  myocardial infarction and complex morphologies,  such as aorto-ostial, unprotected left main lesion,  chronic total occlusion, graft, thrombosis, or a heavily calcified or extremely tortuous lesion. | Under the age of 18 or older than 80 years old  Patient is a woman who is pregnant or nursing  Known contraindications to medications such as heparin, antiplatelet drugs, or contrast  Currently participating in another trial and not yet at its primary endpoint  Concurrent medical condition with a life expectancy of less than 1 years  Required long-term oral anticoagulant therapy  Cardiogenic shock  Previous stent implantation within 6 months  In-stent thrombosis | Exclusion criteria were need for oral anticoagulation or  antiplatelet therapy other than aspirin and P2Y12 receptor  blockers, history of intracranial bleeding, and known intolerance to clopidogrel. | 1. Patients >80 years 2. Increased risk of bleeding, anemia, thrombocytopenia 3.A need for oral anticoagulation therapy  4. Pregnant women or women with potential childbearing  5.Life expectancy <1 year | Exclusion criteria were stroke within 3 months or any permanent neurological  deficit; any previous intracranial bleed or intracranial  disease (eg, aneurysm or fistula); previous coronary  artery bypass graft surgery; any planned surgery within  12 months; any reason for which antiplatelet therapy  might need to be discontinued within 12 months;  severe chronic kidney disease (defined as an estimated  glomerular filtration rate <20 mL/min per 1·73 m2); need  for chronic oral anticoagulation (ie, warfarin or coumadin  or direct oral anticoagulants); a platelet count of less than  100 000 mm3; contraindication to aspirin or ticagrelor;  liver cirrhosis; people intending to become pregnant; a  life expectancy of less than 1 year; and any condition  likely to interfere with study processes, including  medication compliance or follow-up visits (eg, dementia,  alcohol abuse, severe frailty, or required to travel a long  distance for follow-up visits). |
| P2Y12i Used | Ticagrelor | Clopidogrel | Clopidogrel | Ticagrelor/Aspirin | Clopidogrel/Prasugrel; Prasugrel switched to clopidogrel after 1 month | Ticagrelor | Ticagrelor; Replaced with clopidogrel if dyspnea persisted |
|  |  |  |  |  |  |  |  |
|  |  |  |  |  |  |  |  |
| Age, y | 64.6 ± 10.3 | 76.0 ± 8.74 | 67 ± 10 | N/A | 68.6 ± 10.7 | 61 ± 10 | 62 ± 8 |
| Male, n (%) | 12,254 (76.7) | 3,171 (69.3) | 2,087 (69.1) | N/A | 2,337 (77.7) | 2,374 (83.3) | 2,521 (74.1) |
| Hypertension, n (%) | 11,715 (73.4) | 3,553 (77.6) | 2,009 (66.5) | N/A | 2,221 (73.8) | 1,348 (47.3) | 2,121 (62.4) |
| Diabetes mellitus, n (%) | 4,038 (25.3) | 1,538 (33.6) | 1,135 (37.6) | N/A | 1,159 (38.5) | 830 (29.1) | 1,075 (31.6) |
| CKD | — | 876 (19.1) | 408 (13.5) | N/A | 166 (5.5) | 222 (7.8) | 248 (7.3) |
| Dyslipidemia, n (%) | 10,768 (67.4) | 3,097 (67.6) | 2,454 (81.2) | N/A | 2,244 (74.6) | 2,106 (73.9) | 2,335 (68.7) |
| Current smoker, n (%) | 4,169 (26.1) | 414 (9.1) | 500 (16.6) | N/A | 710 (23.6) | 1,094 (38.4) | 968 (28.5) |
| Ischemic Stroke, n (%) | 421 (2.6) | 410 (9.0) | 201 (6.7) | N/A | 186 (6.2) | 92 (3.2) | 301 (8.9) |
| Previous Bleeding, n (%) | 98 (0.6) | 359 (7.8) | **—** | N/A | 47 (1.6) | — | — |
|  |  |  |  |  |  |  |  |
|  |  |  |  |  |  |  |  |
| STEMI, n (%) | 2,092 (13.1) | 538 (11.7) | 88 (2.9) | N/A | 561 (18.6) | 1,150 (40.4) | 948 (27.9) |
| NSTEMI, n (%) | 3,373 (21.1) | 1,153 (25.2) |  | N/A | 180 (6.0) | 992 (34.8) | 1,076 (31.6) |
| UA, n (%) | 2,022 (12.6) | 520 (11.4) | 1,104 (36.6) | N/A | 407 (13.5) | 708 (24.8) | 1,376 (40.5) |
| CCS, n (%) | 8, 481 (53.1) | 1849 (40.4) | 1,828 (60.5) | N/A | 1861 (61.8) | — | — |
| Prior MI, n (%) | 3,710 (23.2) | 864 (18.9) | 108 (3.6) | N/A | 406 (13.5) | 52 (1.8) | 299 (8.8) |
| Prior PCI, n (%) | 5,221 (32.7) | 1,188 (25.9) | 521 (17.3) | N/A | 1,032 (34.3) | 184 (6.5) | 345 (10.1) |
| NACE, (Y/N) | N | Y | Y | Y | Y | Y | Y |
| MACCE, (Y/N) | Y | Y | Y | Y | Y | Y | Y |
| MCRB, (Y/N) | Y | Y | Y | Y | Y | Y | Y |
| All-cause death, (Y/N) | Y | Y | Y | N/A | Y | Y | Y |
| CV death, (Y/N) | Y | Y | Y | N/A | Y | Y | Y |
| Stent thrombosis | Y | Y | Y | N/A | Y | Y | Y |
| MI | Y | Y | Y | N/A | Y | Y | Y |
| TVR | Y | Y | Y | N/A | Y | Y | Y |
| Stroke | Y | Y | Y | N/A | Y | Y | Y |

Abbreviations: m, mean; SD, standard deviation; n, number of events; %, percentage event; N/A, not available; NR, not reported; N, no; Y, yes; MI, myocardial infarction; NSTEMI, non ST-elevation MI; STEMI, ST-elevation MI; UA, unstable angina; CCS, chronic coronary syndrome; PCI, percutaneous coronary intervention; RCA, right coronary artery; RCx, right circumflex artery; CKD, chronic kidney disease; NACE, net adverse clinical events; MACCE, major adverse cardiovascular or cerebrovascular events; CRB, clinically relevant bleeding; TVR, target vessel revascularization;
